# Porous Collagen Scaffold Reinforced with Surfaced Activated PLLA Nanoparticles

**DOI:** 10.1100/2012/695137

**Published:** 2012-02-01

**Authors:** Cancan Xu, Wei Lu, Shaoquan Bian, Jie Liang, Yujiang Fan, Xingdong Zhang

**Affiliations:** National Engineering Research Center for Biomaterials, Sichuan University, Sichuan, Chengdu 610064, China

## Abstract

Porous collagen scaffold is integrated with surface activated PLLA nanoparticles fabricated by lyophilizing and crosslinking via EDC treatment. In order to prepare surface-modified PLLA nanoparticles, PLLA was firstly grafted with poly (acrylic acid) (PAA) through surface-initiated polymerization of acrylic acid. Nanoparticles of average diameter 316 nm and zeta potential −39.88 mV were obtained from the such-treated PLLA by dialysis method. Porous collagen scaffold were fabricated by mixing PLLA nanoparticles with collagen solution, freeze drying, and crosslinking with EDC. SEM observation revealed that nanoparticles were homogeneously dispersed in collagen matrix, forming interconnected porous structure with pore size ranging from 150 to 200 *μ*m, irrespective of the amount of nanoparticles. The porosity of the scaffolds kept almost unchanged with the increment of the nanoparticles, whereas the mechanical property was obviously improved, and the degradation was effectively retarded. *In vitro* L929 mouse fibroblast cells seeding and culture studies revealed that cells infiltrated into the scaffolds and were distributed homogeneously. Compared with the pure collagen sponge, the number of cells in hybrid scaffolds greatly increased with the increment of incorporated nanoparticles. These results manifested that the surface-activated PLLA nanoparticles effectively reinforced the porous collagen scaffold and promoted the cells penetrating into the scaffold, and proliferation.

## 1. Introduction

Tissue engineering, which utilizes biodegradable scaffolds, cells, and cell factors to construct three-dimensional (3-D) engineered tissues for *in vivo* implantation, provides a promising remedy for the treatment of tissue defect and has been largely developed in recent years [1–5]. The tissue engineering approaches involve isolating and expanding cells *in vitro*, seeding and culturing the expended cells in appropriate scaffold to construct the engineered tissue, and transplanting *in vivo* [6–8].

Scaffolds provide framework and initial support for cell adhesion, proliferation, differentiation, extracellular matrix secretion, and tissue formation in tissue engineering technology. Therefore, the fabrication of biocompatible scaffold is one of the most crucial aspects in tissue engineering [9–13]. Both the synthetic biodegradable polymers (PLLA, PGA, PLGA, PCL, etc.) [14–18] and the naturally derived polymers (collagen, silk fibroin, chitosan, cellulose, etc.) [19–23] have been fabricated as scaffolds to guide the regeneration of cartilage, bone, skin, ligament, bladder, and liver.

Employing collagen as the scaffold materials in tissue engineering applications had been numerously reported because of its good cytocompatibility [24–28]. Nevertheless, the fast degradation and mechanical weakness still remain issues in their application as tissue scaffolds.

Combination of collagen with synthetic polymers led to improved cell adhesion and benefited the cell proliferation while maintaining their good mechanical strength [29–35]. Hiraoka et al. reported the hybrid scaffolds which were fabricated by dispersing PLLA fibers in collagen matrix and then freeze drying [36]. These scaffolds kept the interconnected porous structure of collagen sponge. Meanwhile, their compression strength was significantly enhanced because of PGA fiber incorporation. Proliferation of fibroblast in the fiber-reinforced scaffold was much better than that in the collagen sponges without fibers, suggesting the importance of mechanical properties on the cellular behavior. Liu et al. activated the PLA fiber surface by aminolyzation of PLA with hexanediamine and employed the surface-activated PLA fibers for the reinforcement of collagen scaffold [37]. Compared with untreated PLA fiber, the surface-activated fibers provided the scaffolds with higher compression modulus. Thanks to the improved mechanical property, better cell seeding and proliferation were also observed in the scaffolds with surface-activated PLA fibers than that with untreated PLA fibers.

Besides the fibrous polymeric filler, microspheres of biodegradable polymer were also used to improve the mechanical properties of porous scaffold. Hong et al., reported a hydrogel composite scaffold integrated with collagen-coated polylactide microcarriers for cartilage regeneration [38]. Collagen coating on surface of PLA microspheres improved both the cell attaching property of the microspheres and the interaction between the microspheres and the natural chitosan matrix. Therefore, greatly improved mechanical properties were achieved when high microsphere contents were used.

These hybrid materials combined the high strength of synthesized biodegradable polymer and the good biocompatibility and bioaffinity of natural collagen, providing a useful approach for the development of scaffold materials. In addition, the cell proliferation in these hybrid scaffolds was generally found to be much better than that in the sponges without synthetic polymer fillers [39].

In this research, a porous hybrid scaffold comprised of collagen and surface-modified PLLA nanoparticles was prepared. The PLLA nanoparticles were chemically modified by surface-initiated polymerization of acrylic acid to introduce carboxylic groups on the surface, which ensured the homogeneous dispersion and chemical combination of the nanoparticles within the collagen matrix. Compare with other forms of synthesized polymer fillers, such as fibers and microspheres, incorporation of PLLA nanoparticles in collagen greatly improved the mechanic properties of the hybrid scaffolds and obtained controllable degradation behavior without any apparent alteration to their morphology. The cell adhesion and proliferation in the hybrid scaffolds were found to be largely promoted.

## 2. Materials and Methods

### 2.1. Materials

PLLA (*M*_*w*_ = 200,000), fluorescein diacetate (FDA), and rhodamine 6G were purchased from Sigma. Co. Ltd. N-Hydroxysuccinimide (NHS) and acrylic acid (AA) were obtained from Chengdu Kelong Chemical Company, China. 1-ethyl-(3-dimethylaminopropyl) carbodiimide hydrochloride (EDCI) was purchased from Astatech (Chengdu) Pharmaceutical Co. Ltd. Collagen Type I was harvested from bovine skin by pepsin treatment in acetic acid as described in the previous report [37]. Cell culture medium (PRMI-1640) and fetal bovine serum (FBS) were products of Hyclone Biology Company, German. AA was distilled under vacuum (100 pa, 40°C) before used. All other reagents were used as received without further purification.

### 2.2. Preparation of Surface-Modified PLLA Nanoparticles

At first, PLLA microspheres (smaller than 105 *μ*m) were prepared employing an emulsion solvent evaporation technology. Briefly, a PLLA solution (5 g in 250 mL dichloromethane) was dropped into 1.6 L methanol under stirring. The mixture was stirred for 18 h (1000 rpm) at room temperature to evaporate most of the organic solvent. Afterwards, deionized water was added to replace the residual organic solvent. After 3 times decantation and water replacement, the mixture was lyophilized. PLLA microspheres with diameters smaller than 105 *μ*m were obtained by sieving.

Then, PLLA microspheres were dispersed in hydrogen peroxide (H_2_O_2_) in a glass culture dish and irradiated under UV light (3000 × 100 *μ*J/cm^2^) for 4 h to introduce –OOH groups onto their surface, washed with deionized water and vacuum dehydrated immediately. Subsequently, photo-oxidized PLLA microspheres (700 mg) and fresh distilled AA solution (10 v%, 175 mL) were added into a flask equipped with a magnetic stirrer, and then degassed by repeated vacuum and Ar flux. FAS (0.015 M, vacuumed and purged with Ar in advance) was injected into the flask and the reaction was kept at 37°C with Ar flux for 1 hour to allow the surface –OOH initiated polymerization of AA. The microspheres were collected by centrifugation (3000 rpm for 15 min), vigorously washed with deionized water and ethanol to remove the unreacted monomer, and then lyophilized.

The nanoparticles were prepared using modified PLLA by the dialysis method. 100 mg of modified PLLA microspheres were dissolved in 20 mL of tetrahydrofuran (THF) and vacuum filtrated. The filtration was dialyzed thoroughly against deionized water, and the nanoparticles were obtained by lyophilizing.

### 2.3. Characterization of the Microspheres and Nanoparticles

The density of the peroxide groups on the surface of the photooxidized PLLA microspheres was measured by iodometry method. 5 mg of photooxidized PLLA microspheres and 50 mg KI were added into a color comparison tube and dispersed with 5 mL isopropanol. Subsequently, a drop of trichloride ferric solution (100 ppm) was added, and the volume of the mixture was metered to 10 mL with isopropanol. After incubated at 60°C for 10 min, the colorless mixture turned yellow, and the appearance of the yellow color was measured in a spectrophotometer at 360 nm using stoppered crystal cells. Benzoyl peroxide was used for the construction of calibration curve.

The density of the –COOH groups grafted on the PLLA microsphere surface was determined by a modified rhodamine-carboxyl interaction method according to the published research [29].

The zeta potential, diameter and size distribution of the nanoparticles were determined by dynamic light scattering (DLS, Malvern Nano-ZS). The morphology of the PLLA nanoparticles was observed by SEM (Hitachi S-4800, Japan).

### 2.4. Preparation of Porous Collagen Scaffold Integrated with PLLA Nanoparticle

The porous collagen scaffolds integrated with PLLA nanoparticles were prepared by lyophilization technique. 0.5 g of collagen was dissolved in 40 mL acetic acid solution (3 v%, pH = 2.8). Then, pH of the collagen solution was gently adjusted to 7.4 with sodium hydroxide solution (1 M). Afterwards, PLLA nanoparticles dispersed in deionized water were added under gentle stirring. The final weight ratio of the collagen (Col) was 6 mg/mL, and the weight of nanoparticles (NP) was controlled as 1, 3, and 6 mg/mL (Col : NP = 6 : 0, 6 : 1, 6 : 3 and 6 : 6 (w : w)), respectively. The whole procedure was conducted on an ice bath to prevent the gelation of collagen. Finally, the mixtures were injected into 48 well culture plate, frozen at −20°C and lyophilized. For crosslinking, lyophilized scaffolds were further vacuum dehydrated (100 pa, 110°C for 48 h), soaked in 95 v% ethanol solution containing 50 mM EDC and 50 mM NHS under vacuum (100 pa for 30 min), incubated at room temperature for 48 h, washed with deionized water repeatedly and lyophilized. 

To observe the inner side of the scaffolds, they were crosscut into pieces from the middle with a razor blade and coated with gold before being observed by SEM (S-4800, Hitachi, Japan).

### 2.5. Porosity

The porosity (*P*) of the scaffolds was measured by ethanol infiltration method. Briefly, weighed scaffolds (*W*_0_) were soaked in ethanol under vacuum (100 pa for 20 min) to exhaust the air bubbles. Then the scaffolds were taken out, wiped out the surface ethanol and weighed immediately (*We*).

The porosity of the scaffolds was defined as

(1) $$P=\frac{\left(We-W_{0}\right)}{\left(\rho Vs\right)}\times 100\%.$$

where *ρ* represents the density of ethanol at room temperature (0.789 mg/mL). *Vs* was calculated from the geometry of the scaffolds (height and diameter of the crosssection).

### 2.6. Compressive Modulus

The scaffolds (10 mm height × 10 mm diameter) were soaked in PBS under vacuum (100 pa) for 30 min and then compressed by a mechanical tester (AGS-5D, Shimadzu, Japan) with the crosshead speed at 0.5 mm/min until the elastic deformation of the scaffolds reached 40%. The compressive modulus was determined from the compressive curve.

### 2.7. *In vitro* Degradation

The weight loss ratio of the scaffolds on degradation was monitored as a function of incubation time in PBS at 37°C. Briefly, the scaffolds were cut into pallets (2 mm height × 10 mm diameter), weighed (*W*_0_), and incubated in 1 mL PBS (pH 7.26 and replaced every 2 days) at 37°C. The pallets were taken out at desired intervals, washed repeatedly with deionized water, lyophilized, and weighed (*W*_*d*_). The weight loss ratio of the scaffold was defined as (*W*_0_ − *W*_*d*_)/*W*_0_ × 100%.

### 2.8. Cell Culture *In vitro*

Scaffold samples (2 mm height × 10 mm diameter) were sterilized with 75 v% ethanol solution for 15 min, rinsed with PBS (3 × 10 min), then transferred into 24-well plates, and infiltrated with culture medium in incubator overnight to allow equilibrium swelling. The scaffold pallets were covered with glass rings (*ϕ*12 × 10 mm) to prevent the scaffold floating, and then 100 *μ*L L929 cell suspension at a density of 5 × 10^6^ cells/mL was dropped onto each scaffold pallet. After 6 h, the scaffold pallets were turned over and placed in new wells, and another 100 *μ*L L929 suspension was added. 4 h later, 1 mL medium was added to each well. After 24 h, the glass rings were removed, and the scaffolds were transferred into wells of 12-well culture plate, and then cultured under 5% CO_2_ atmosphere at 37°C. The culture medium was changed every two days.

### 2.9. Cell Distribution and Morphology

Cell distribution in the scaffolds was observed using confocal laser-scanning microscope (CLSM, TCS SP5, Leica, Germany) and SEM. For CLSM observation, the cell-scaffold constructs after 24 h incubation were taken out from the medium, rinsed with PBS, and then immersed into 1 *μ*g/mL FDA solution for 3 min for staining. The viable cells in the scaffolds were visualized under CLSM. For SEM observation, the scaffolds were rinsed with deionized water and fixed in 0.25% glutaraldehyde at 4°C for 24 h. Then, the scaffolds were washed with deionized water, frozen at −80°C for 24 h and lyophilized. To observe the inner side, the lyophilized scaffolds were crosscut at the middle and coated with a thin gold layer.

### 2.10. DNA Assay

DNA contents were determined by fluorescent spectrophotometer using H33258 dye assay [26]. Briefly, cell-scaffold constructs cultured for 1, 3, and 5 days were washed with PBS and deionized water and froze at −80°C. Then, the constructs were lyophilized, digested with papain (0.1%, 1 mL, 24 h at 60°C), and then centrifuged at (10,000 rpm for 3 min at 4°C). For each measurement, 10 *μ*L supernate of the sample was mixed with 2 mL H33258 dye (0.1 *μ*g/mL), and the fluorescent emission value of the mixture was measured by fluorescent spectrophotometer at excitation and emission wavelength of 360 and 460 nm, respectively. The amount of the DNA contents was calculated on the basis of the calibration curve deduced from the foregone calf thymus DNA.

### 2.11. Statistic Analysis

Data were analyzed using one-tailed, standard Student's *t*-tests with *P* < 0.05 indicating statistical significance. Values with *P* < 0.06 were reported as well. Results are reported as mean ± SD.

## 3. Results and Discussion

### 3.1. Preparation and Characterization of Surface-Modified-PLLA Nanoparticle

For the fabrication of porous collagen scaffold integrated with PLLA nanoparticle, it is necessary to homogeneously disperse the nanoparticles in the collagen matrix. However, PLLA is a hydrophobic polymer, thus the nanoparticles derived from PLLA tend to aggregate in aqueous solution. This idiosyncrasy makes the PLLA nanoparticles difficult to mix with the collagen solution equably. In addition, the hydrophobic characteristic of PLLA might result in the weak interaction between PLLA nanoparticles and collagen matrix and thus limited the improvement of the mechanical property.

**Scheme 1:**
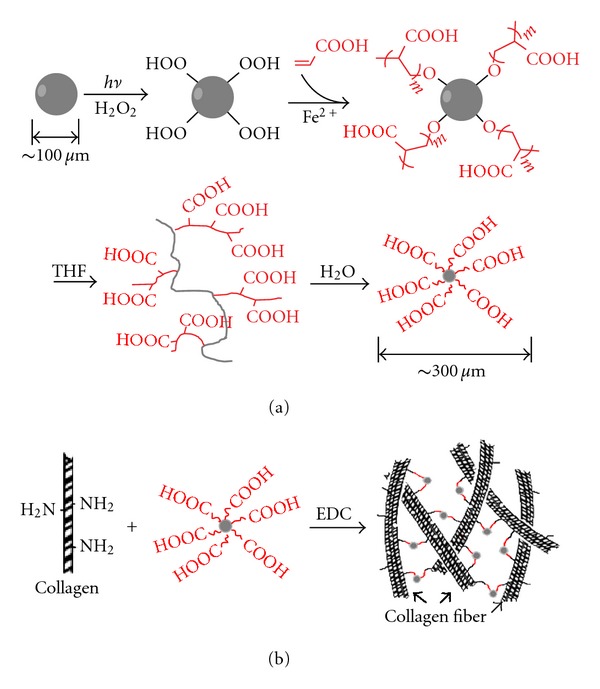
Fabrication of porous collagen integrated with PLLA nanoparticles.

In order to fabricate the PLLA nanoparticles with hydrophilic surface and introduce reactive functional groups, the PLLA was firstly grafted with PAA (Scheme 1(a)). A two-step procedure was employed for graft polymerization of acrylic acid on PLLA [8]. At first, the PLLA was treated by UV radiation in the hydrogen peroxide to introduce active –OOH groups onto PLLA chain. Since this photochemical reaction occurs on the surface, the PLLA was transformed into microspheres with diameter less than 105 *μ*m in order to increase the surface area and the reaction sites. The amount of the peroxide groups on the photooxidized PLLA microspheres was measured by iodometry method as 0.052 mmol/g. Subsequently, the UV-radiated PLLA microspheres were treated by Fe^2+^ to generate free radicals, which then initiated the polymerization of acrylic acid. In this way, poly (acrylic acid) was grafted on PLLA microsphere surface. After the graft polymerization of acrylic acid, the content of carboxyl was determined by rhodamine-carboxyl interaction method as 0.186 mmol/g. The small size of the microspheres provided high specific surface area and thus led to enough grafted poly (acrylic acid) on PLLA. 

**Figure 1:**
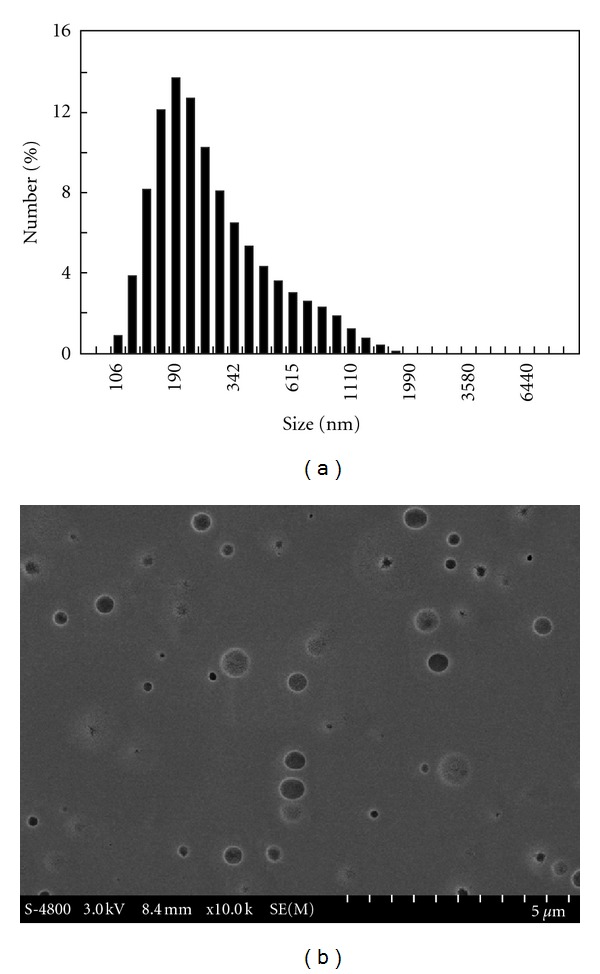
DLS histogram and SEM image of PLLA nanoparticles.

Modified PLLA nanoparticles were fabricated by dialysis method the PLLA solution in THF against water. (Scheme 1(a)) Thanks to the hydrophilic PAA segments, the PLLA did not precipitate on diluting the THF solution with water. In contrast, nanosized particles formed.  Figure 1 showed the morphology and size distribution of the nanoparticles determined by SEM and DLS. The nanoparticles were spheric with average diameter of 316 nm. They showed a negative zeta potential (−39.88 mv), which was attributed to ionization of the carboxyl on the surface of the nanoparticles. The modified PLLA formed stable nanoparticles in water without aggregation even after 2 weeks.

### 3.2. Structure and Physicochemical Properties of Porous Collagen Scaffold Integrated with PLLA Nanoparticle

**Figure 2:**
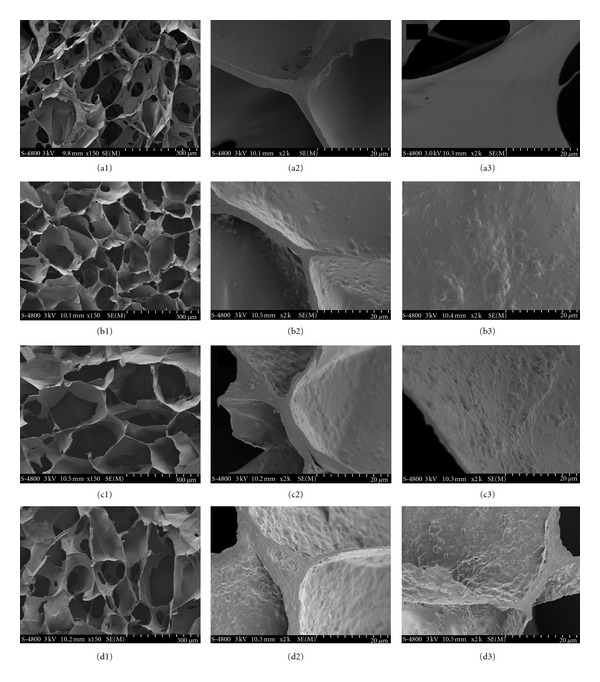
SEM images of porous collagen scaffolds integrated with PLLA nanoparticles. (a1), (a2), (a3): Col : NP = 6 : 0; (b1), (b2), (b3): Col : NP = 6 : 1; (c1), (c2), (c3): Col : NP = 6 : 3; (d1), (d2), (d3): Col : NP = 6 : 6 (w : w).

The porous collagen/PLLA nanoparticle scaffolds were prepared using the common lyophilization method (Scheme 1(b)). PLLA nanoparticles were dispersed in water and then mixed with collagen solution. To prevent the formation of ioncomplex between the negatively charged PAA and the positively charged collagen molecules, which might result in the precipitation of the collagen, acidic collagen solution was neutralized by sodium hydroxide solution before the addition of PLLA nanoparticles. This procedure was conducted under chill condition in order to prevent the gelation of neutralized collagen solution. The nanoparticles were distributed in collagen solution uniformly to form a semitransparent mixture without aggregation and precipitation. Freeze drying and then subsequent crosslinking by EDC treatment resulted in porous scaffold.  Figure 2 illustrated the SEM images of the scaffold with various Col/NP ratios. In despite of the addition of PLLA nanoparticles, the scaffolds kept the same porous microstructures, such as pore size, shape, and interconnection, with the pure collagen sponge. The cross-section of the scaffolds exhibited pore diameters ranging from 150 to 250 *μ*m (Figure 2, (a1), (b1), (c1), and (d1)). However, the thickness of the wall increased with the increment of PLLA nanoparticles (Figure 2, (a2), (b2), (c2), and (d2)). The roughness of the surface also accreted when the ratio of nanoparticles increased (Figure 2, (a3), (b3), (c3), and (d3)). It can be seen that the distribution of the nanoparticles in collagen was substantially homogeneous, and nanoparticles were completely embedded in collagen matrix. The outer surface of the pore wall composed of collagen matrix without unmasked PLLA nanoparticles, providing the scaffolds with properties suitable for cell adhesion and proliferation.

**Figure 3:**
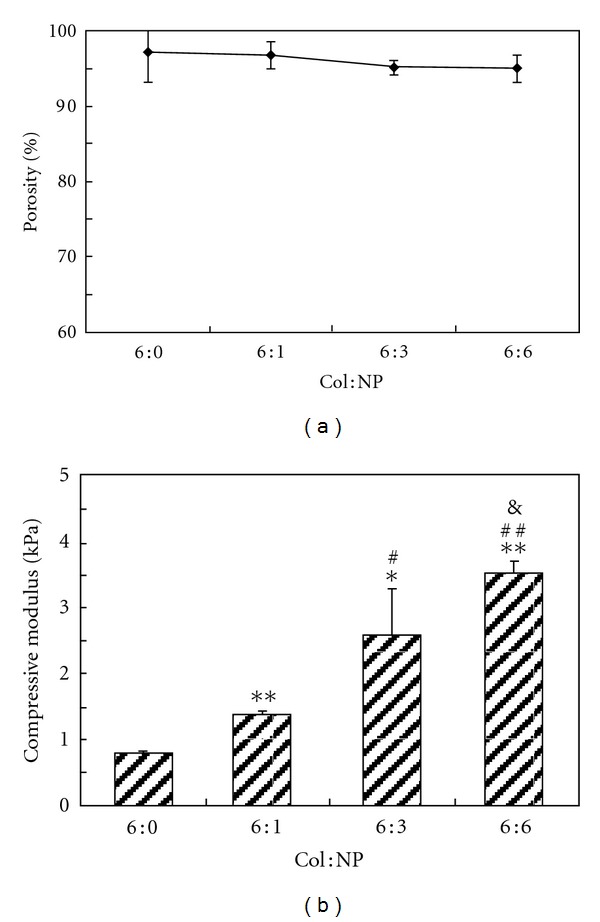
Porosity and compress modules of porous collagen scaffolds integrated with PLLA nanoparticles. (a) Porosity (b) compress modules. *n* = 3, *P < 0.05, **P < 0.01 (compared to pure collagen sponges with Col : NP = 6 : 0 (w : w)); ^#^ *P* < 0.05, ^##^ *P* < 0.01 (compared to scaffolds with Col : NP = 6 : 1 (w : w)), and *P* < 0.06 (compared to sponges with Col : NP = 6 : 3 (w : w)).

Figure 3 showed the porosity and compressive modulus of scaffolds with different Col : NP (w : w) ratio. The porosity of the scaffolds decreased from 98% (pure collagen sponge, Col : NP = 6 : 0) with the incorporation of PLLA nanoparticles. However, it is worth to note that the decrease of porosity was very slight. Even integrating equivalent amount of PLLA nanoparticles (Col : NP = 6 : 6), the porosity still kept over 95% (Figure 3(a)). The high porosity of the scaffolds is admirable for cell culture in allowing maximum adhesion and proliferation of cells, leaving space for newly synthesized matrix via a large surface: volume ratio [7, 23].

Incorporation of PLLA nanoparticles enabled collagen scaffold to significantly increase the compression modulus compared with the sponge without PLLA nanoparticles. The compression modulus of the scaffolds increased from 0.78 kpa for pure collagen (Col : NP = 6 : 0 (w : w)) to 1.3, 2.5, and 3.5 kpa for hybrid scaffolds with Col : NP = 6 : 1, 6 : 3, 6 : 6 (w : w), respectively. This can be ascribed to both the physical filling effect of nanoparticles and the chemical bond between the nanoparticles and collagen matrix. The PAA on the surface of nanoparticles can chemically react with collagen macromolecule, so that the chemically bond makes strong interaction between the nanoparticles and collagen and results in higher mechanical property. The reinforcement of the collagen scaffolds is conspicuous and can be easily controlled by adjusting the incorporated amount of PLLA nanoparticles.

**Figure 4:**
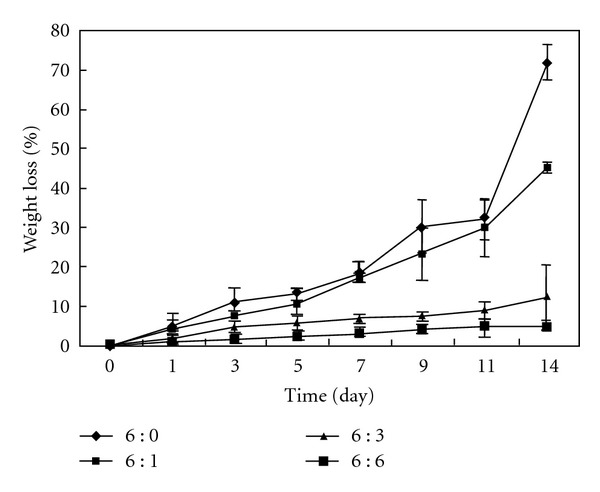
Degradation behavior of porous collagen scaffolds integrated with PLLA nanoparticles.

Figure 4 showed the weight loss profile of the scaffolds upon incubation in PBS buffer at 37°C. Obviously, the integration of PLLA nanoparticles had distinct effects on inhibiting the fast degradation of collagen scaffolds. The weight loss of pure collagen sponge reached 71% after 14 days incubation, whereas great slowdown was observed with the integration of nanoparticles (45%, 12%, and 5% for scaffolds of Col : NP = 6 : 0, 6 : 1, 6 : 3, and 6 : 6 (w : w), resp.). The slow degradation of the composite scaffolds might be described to the crosslinking between collagen and the carboxylic groups on the surface of nanoparticles. Degradation rate of the scaffold materials has greatly effect on the adhesion, growth, and proliferation of cells and further affects the formation of the new engineered tissue. The weight loss results of the hybrid scaffolds suggested that the integration of PLLA nanoparticles is a useful way to adjust the degradation rate of the collagen scaffolds. 

### 3.3. Cell Seeding and Culture *In vitro*

**Figure 5:**
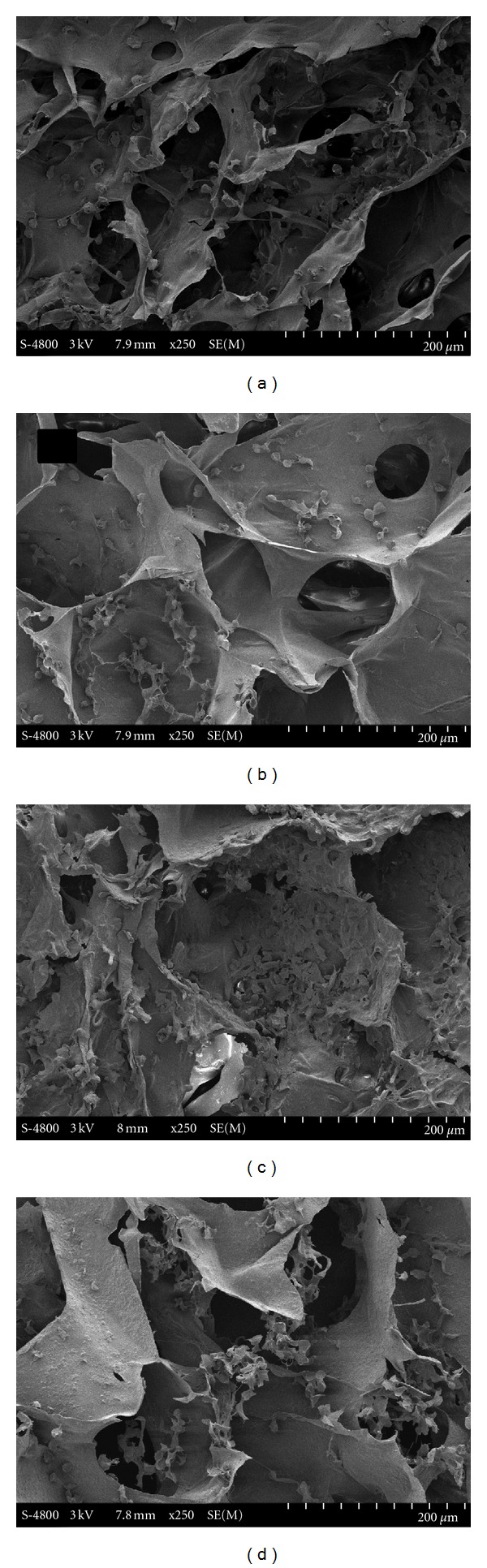
Cross-sectional SEM images of L929 fibroblast cells cultured in the scaffolds. (a) Col : NP = 6 : 0; (b) Col : NP = 6 : 1; (c) Col : NP = 6 : 3; (d) Col : NP = 6 : 6 (w : w).

Figure 5 showed the cross-sectional SEM photographs of L929 cells cultured on the hybrid scaffolds 24 h after cell seeding. The seeded L929 cells were homogeneously distributed inside the scaffolds along the pore walls, in shape of roundness. Because the PLLA nanoparticles were completely covered by collagen, the adhesion of cells on the pore walls was not obviously different between the scaffolds with different Col : NP ratio. Nevertheless, although not quantitatively, the number of cells seemed to increase with the increment of the integrated nanoparticles.

**Figure 6:**
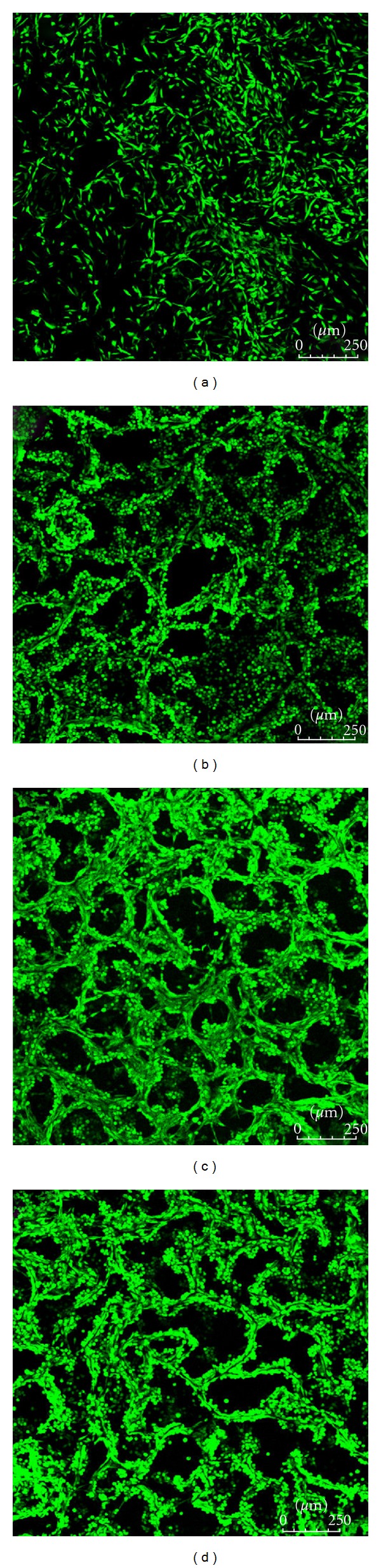
CLSM images of L929 fibroblast cells cultured in the hybrid scaffolds. (a) Col : NP = 6 : 0; (b) Col : NP = 6 : 1; (c) Col : NP = 6 : 3; (d) Col : NP = 6 : 6 (w : w).

Figure 6 showed the CLSM images of the viable cells in hybrid scaffolds after cultures for 24 h. L929 cells infiltrated into all the collagen scaffolds containing various amount of nanoparticles. It was clearly shown that the cells had attached to the pore walls, and most of the adhesive cells had expanded their antennae. The porous microstructure of the scaffolds was visualized by the adhesive cells. The distribution of cells was homogeneous in all the sponges, but the numbers of cells increased with the increment of the integrated PLLA nanoparticles.

**Figure 7:**
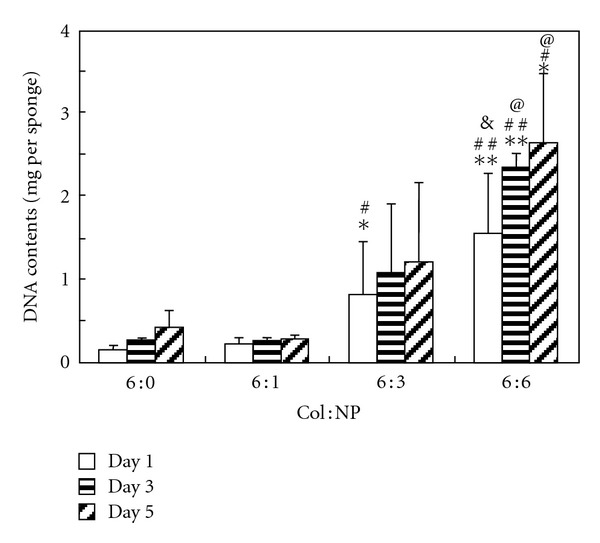
DNA contents of L929 fibroblast cells in the hybrid scaffolds. *n* = 4, *P < 0.05, **P < 0.01 (compared to sponges with Col : NP = 6 : 0 (w : w) at day1, day3, or day5, resp.), ^#^ *P* < 0.05, ^##^ *P* < 0.01 (compared to sponges with Col : NP = 6 : 1 (w : w) at day1, day3, or day5, resp.), ^&^ *P* < 0.06, ^@^ *P* < 0.05 (compared to sponges with Col : NP = 6 : 3 (w : w) at day1, day3, or day5, resp.).

Figure 7 showed the DNA contents of L929 cells cultured in the scaffolds for 1, 3, and 5 days, respectively. In agreement with the CLSM observation, after 1-day culture, the DNA contents in scaffolds greatly increased with the increment of nanoparticles. This finding demonstrated that the cell seeding efficiency in the scaffolds could be markedly improved by the incorporation of the nanoparticles in the scaffolds. The nanoparticles might contribute from two aspects for the improving seeding efficiency. Firstly, the intensification of the scaffolds increased resistance to compression when cells are seeded, so the volume available for cell penetrating was larger. Secondly, increased roughness of the pore walls and the collagen surface provided a more biocompatible environment for cell attachment and proliferation.

With the elongation of culture time, the DNA contents in the scaffolds significantly increased, but the proliferation rates were different in scaffolds with different amount of nanoparticles. It can be reckoned that, in pure collagen sponge, the DNA content increased about 2 times, whereas, in hybrid scaffold (Col : NP = 6 : 6), it increased only less than 1 time. The relatively lower proliferation rate in hybrid sponges might be due to the higher initially seeded cell number. The initial cell number in the hybrid scaffold (Col : NP = 6 : 6 (w : w)) is about 9 times higher than that in the pure collagen sponge. In the limited interspace of the pores, the cell growth might be greatly influenced by the rather high cell density. Comparing the hybrid scaffolds with pure collagen sponge, it can be found that the absolute DNA contents in the hybrid scaffolds were much higher than that in the pure collagen sponge. Even after 5-day culture, the DNA contents in pure collagen were less than the initial DNA content in hybrid sponge (Col : NP = 6 : 3 (w : w)).

## 4. Conclusion

Porous collagen scaffolds integrated with PLLA nanoparticles were fabricated by lyophilizing and crosslinking via EDC treatment. Surface-modified PLLA nanoparticles can be homogeneously dispersed in and chemically bonded to collagen matrix. The integration of PLLA nanoparticles greatly promoted the mechanical properties and slowed down the degradation of the scaffolds, while kept their interconnected porous microstructure, such as average pore size and porosity. *In vitro* cell seeding and culture studies revealed that cells infiltrated into the scaffolds and distributed homogeneously. The proliferation of cells in the hybrid scaffold increased with the increment of incorporated nanoparticles. These results demonstrated that incorporation of nanosized particles into collagen matrix might be a useful approach for the development of the tissue engineering scaffold, and the porous collagen scaffold integrated with PLLA nanoparticles might be a promising biocompatible scaffold for tissue engineering.
